# Анализ оказания медицинской помощи с применением телемедицинских технологий в ГНЦ РФ ФГБУ «НМИЦ эндокринологии»

**DOI:** 10.14341/probl13500

**Published:** 2024-11-04

**Authors:** А. С. Назарова, С. С. Приказчикова, В. Ю. Калашников, Г. А. Мельниченко, Н. Г. Мокрышева

**Affiliations:** Национальный медицинский исследовательский центр эндокринологии; Национальный медицинский исследовательский центр эндокринологии; Национальный медицинский исследовательский центр эндокринологии; Национальный медицинский исследовательский центр эндокринологии; Национальный медицинский исследовательский центр эндокринологии

**Keywords:** телемедицина, эндокринология, Координационный совет, организация здравоохранения

## Abstract

**ОБОСНОВАНИЕ:**

ОБОСНОВАНИЕ. В первые месяцы после пандемии новой коронавирусной инфекции оказание медицинской помощи посредством телемедицинских технологий занимало лидирующие позиции, в частности по поводу эндокринных нозологий. Между тем в настоящее время всеобъемлющей информации о телекоммуникационном взаимодействии между врачами различных медицинах организаций субъектов Российской Федерации и сотрудников федеральных центров недостаточно, что обуславливает актуальность изучения данной темы.

**ЦЕЛЬ:**

ЦЕЛЬ. Анализ оказания медицинской помощи при дистанционном взаимодействии медицинских работников с применением телемедицинских технологий («врач-врач») между ГНЦ РФ ФГБУ «НМИЦ эндокринологии» и субъектами Российской Федерации в 2019–2023 гг.

**МАТЕРИАЛЫ И МЕТОДЫ:**

МАТЕРИАЛЫ И МЕТОДЫ. Было проведено одноцентровое обсервационное одномоментное одновыборочное исследование. В исследование включали завершенные плановые и неотложные телемедицинские консультации (ТМК) формата «врач-врач» по профилям «эндокринология» и «детская эндокринология», проведенные через телемедицинскую систему дистанционных консультаций федерального и регионального уровней с 2019–2023 гг. Анализировались данные обращаемости медицинских сотрудников субъектов Российской Федерации (РФ) за ТМК в ГНЦ РФ ФГБУ «НМИЦ эндокринологии» (ежегодная динамика обращаемости, частота запросов каждого региона РФ, нозологическая структура запросов, число госпитализированных в ГНЦ РФ ФГБУ «НМИЦ эндокринологии» пациентов по итогам проведенных телемедицинских консультаций, анализ межинститутского взаимодействия ГНЦ РФ ФГБУ «НМИЦ эндокринологии» с другими федеральными центрами).

**РЕЗУЛЬТАТЫ:**

РЕЗУЛЬТАТЫ. В период с 2019–2023 гг. было проведено 14 475 телемедицинских консультаций «врач-врач». В 2019 г. в ГНЦ РФ ФГБУ «НМИЦ эндокринологии» обратились медицинские работники из 78 субъектов Российской Федерации. К 2023 г. данный показатель возрос до 88 (включая вновь присоединенные территории). Регионами-лидерами по обращаемости врачей в ГНЦ РФ ФГБУ «НМИЦ эндокринологии» являлись Ямало-Ненецкиий автономный округ, Тамбовская область и Астраханская область. В нозологической структуре наибольшее количество консультаций у взрослых пациентов было проведено по поводу акромегалии и первичного гиперпаратиреоза, у детей — по поводу низкорослости и сахарного диабета 1 типа. В период с 2019-2023 гг. ГНЦ ФГБУ «НМИЦ эндокринологии» направил суммарно 300 «исходящих» запросов в 17 медицинских организаций.

**ЗАКЛЮЧЕНИЕ:**

ЗАКЛЮЧЕНИЕ. Консультирование с применение телемедицинских технологий стало удобным и многофункциональным способом оказания медицинской помощи. Дальнейший анализ места телемедицины в практическом здравоохранении позволит расширить ее возможности, в том числе рассмотреть вопрос внедрения финансирования территориальным фондом ОМС для данного направления медицинской деятельности.

## Abstract (en)

**BACKGROUND:**

BACKGROUND: In the first months after the pandemic of the COVID-19, the provision of medical care through telemedicine technologies took a leading position, in particular regarding endocrine nosologies. Meanwhile, at present, comprehensive information on telecommunications interaction between doctors of various medical organizations of the regions of the Russian Federation and employees of federal centers is insufficient, which determines the relevance of studying this topic.

**AIM:**

AIM: Analysis of the provision of medical care in remote interaction of medical workers using telemedicine technologies («doctor-doctor») between the Endocrinology Research Centre and the regions of the Russian Federation in 2019–2023.

**MATERIALS AND METHODS:**

MATERIALS AND METHODS: A single-center, observational, single-sample study was conducted, including completed planned and urgent telemedicine consultations in the format of «doctor-doctor « in the fields of «endocrinology» and «pedia­tric endocrinology», conducted through a telemedicine system for remote consultations at federal and regional levels between 2019 and 2023. Data on the referral rate of medical personnel from the regions of the Russian Federation to the Endocrinology Research Centre for telemedicine consultations were analyzed, including annual dynamics of treatment, frequency of referrals from each region, nosological structure of referrals, and number of patients admitted to the center based on the results of these consultations. Analysis of interdepartmental cooperation between the Endocrinology Research Centre and other federal centers was also performed.

**RESULTS:**

RESULTS: In the period from 2019–2023, 14,475 telemedicine consultations «doctor-doctor» were conducted. In 2019, medical workers from 78 regions of the Russian Federation applied to the Endocrinology Research Centre. By 2023, this figure has increased to 88 (including newly annexed territories). The Yamalo-Nenets Autonomous Okrug, Tambov Region and Astrakhan Region were the leading regions in terms of the circulation of medicines at the Endocrinology Research Centre. In the nosological structure, the largest number of consultations in adult patients were conducted for acromegaly and primary hyperparathyroidism, in children for stunting and type 1 diabetes mellitus. In the period from 2019–2023, the Endocrinology Research Centre sent a total of 300 «outgoing» requests to 17 medical organizations.

**CONCLUSION:**

CONCLUSION: Consulting with the use of telemedicine technologies has become a convenient and multifunctional way of providing medical care. Further analysis of the place of telemedicine in practical healthcare will expand its capabilities, including considering the introduction of financing by the territorial fund of compulsory medical insurance for this area of medical activity.

## Обоснование

Эпидемиологическая обстановка во время пандемии COVID-19 навсегда изменила медицинскую помощь. К привычному очному общению врача и пациента присоединился компьютер. Началась эра телемедицинских технологий, а с ней появилась и доступность медицинской помощи для пациентов различного профиля, в том числе с эндокринопатиями. Дистанционные технологии дали жителям сельской местности и удаленных территорий возможность быть на связи с лечащим врачом, а врачам обращаться за помощью к коллегам для решения сложных клинических задач. Телемедицина не только упрощает транспортную логистику для пациента, но и снижает финансовые затраты [1]. Телемедицина стала эффективным инструментом получения медицинской помощи высококвалифицированных специалистов для различных слоев населения.

В первые месяцы после пандемии COVID-19 именно эндокринология занимала лидирующие позиции по использованию телемедицинской помощи [2]. Специфика специальности позволяет сократить число очных визитов к врачу. С помощью дистанционных консультаций стало возможным осуществлять динамическое дистанционное наблюдение пациента, проводить оценочные тесты, включая клинико-лабораторные анализы, назначать и корректировать терапию, оценивать компенсацию углеводного обмена [3].

В Российской Федерации (РФ) порядок оказания медицинской помощи с применением телемедицинских технологий регламентируется Приказом Министерства здравоохранения РФ от 30 ноября 2017 г. №965н. Документ отражает основные аспекты легитимного проведения дистанционных консультаций [4].

В ГНЦ РФ ФГБУ «НМИЦ эндокринологии» (Центр) консультирование с применением телемедицинских технологий по профилям «эндокринология» и «детская эндокринология» началось в 2018–2019 гг. и продолжается в настоящее время. Телемедицина в Центре развивается и функционирует в двух направлениях: «врач-врач» (консультации для медицинских работников из регионов РФ, в том числе в рамках «второго мнения») и «врач-пациент» (консультации для пациентов, наблюдавшихся в Центре ранее — амбулаторно или стационарно).

Работа курируется отделом по вопросам телекоммуникационного взаимодействия с учреждениями субъектов РФ третьего уровня, включая дистанционный и выездной мониторинг, входящим в структуру Координационного совета, в рамках которого осуществляется организационно-методическая поддержка и координация работы эндокринной службы в субъектах РФ.

В течение 5 лет Центром проведено более 15 000 телемедицинских консультаций (ТМК) с регионами РФ («врач-врач»). Сегодня в ГНЦ РФ ФГБУ «НМИЦ эндокринологии» обращаются за помощью врачи практически из каждого уголка России. Расширяются способы проведения ТМК. Стало возможным создание консилиумов и консультирование с использованием видеоконференцсвязи, описание инструментальных исследований и гистосканов в рамках работы «второго мнения».

Накоплены значительные данные по проведению дистанционных консультаций «врач-врач». Опыт работы Центра, как федерального учреждения экспертного уровня по эндокринологии является ценным в рамках развития телемедицинских технологий направления «врач-врач».

## ЦЕЛЬ ИССЛЕДОВАНИЯ

Целью данной работы является анализ оказания медицинской помощи при дистанционном взаимодействии медицинских работников с применением телемедицинских технологий (формат «врач-врач») между ГНЦ РФ ФГБУ «НМИЦ эндокринологии» и субъектами Российской Федерации в 2019–2023 гг.

## МАТЕРИАЛЫ И МЕТОДЫ

## Место и время проведения исследования

Место проведения. ГНЦ РФ ФГБУ «НМИЦ эндокринологии» Минздрава России.

Время исследования. Январь 2019–декабрь 2023 г.

## Изучаемые популяции (одна или несколько)

В исследование были включены завершенные плановые и неотложные телемедицинские консультации формата «врач-врач» по профилям «эндокринология» и «детская эндокринология», проведенные с помощью информационной системы «Телемедицинская система дистанционных консультаций федерального и регионального уровней», доступная по адресу tmk.minzdrav.gov.ru., за указанный период времени. При оформлении запроса пациенты подписывают информированное добровольное согласие на медицинское вмешательство с применением телемедицинских технологий.

## Способ формирования выборки из изучаемой популяции (или нескольких выборок из нескольких изучаемых популяций)

Применялся сплошной способ формирования выборки.

## Дизайн исследования

Одноцентровое обсервационное одномоментное одновыборочное исследование.

## Описание медицинского вмешательства (для интервенционных исследований)

ТМК между ГНЦ РФ ФГБУ «НМИЦ эндокринологии» Минздрава России и субъектами РФ проводились за счет средств федерального бюджета через Телемедицинскую систему Минздрава России согласно действующему Приказу Министерства здравоохранения Российской Федерации от 30 ноября 2017 г. №965н «Об утверждении порядка организации и оказания медицинской помощи с применением телемедицинских технологий» и стандартной операционной процедуры «Порядок проведения телемедицинской консультации врач-врач».

В Центре осуществляются плановые и неотложные консультации с применением телемедицинских технологий. В зависимости от клинической ситуации специалист (врач или ответственное лицо) лечебного учреждения оформляет запрос на консультацию через Федеральную телемедицинскую систему Минздрава России, прилагая медицинскую документацию пациента.

Сотрудник отдела телемедицины ГНЦ РФ ФГБУ «НМИЦ эндокринологии» Минздрава России анализирует полученные данные и направляет их профильному врачу-консультанту Центра. Врач-консультант изучает медицинскую документацию и подготавливает заключение. Отдел по вопросам телекоммуникационного взаимодействия размещает ответ в системе.

## Методы

Проводился анализ данных обращаемости медицинских сотрудников субъектов РФ за ТМК в ГНЦ РФ ФГБУ «НМИЦ эндокринологии» (ежегодная динамика обращаемости, частота запросов каждого региона РФ). Оценивалась нозологическая структура обращаемости (код заболевания по МКБ-10), число госпитализированных в Центр пациентов по итогам проведенных ТМК, анализ межинститутского взаимодействия ГНЦ РФ ФГБУ «НМИЦ эндокринологии» с другими федеральными центрами.

## Статистический анализ

Анализ полученной информации проводился с помощью программ Microsoft Office версии 2016 г.

## Этическая экспертиза

Локальный этический комитет при ГНЦ ФГБУ «НМИЦ эндокринологии» Минздрава России постановил одобрить возможность проведения данной научно-исследовательской работы, выписка из протокола №11 от 13.06.2024 г.

## Результаты

Взаимодействие НМИЦ эндокринологии с регионами в рамках ТМК «врач-врач»

В 2019 г. за ТМК «врач-врач» в ГНЦ РФ ФГБУ «НМИЦ эндокринологии» обратились медицинские работники из 78 субъектов Российской Федерации. Ежегодно число консультируемых регионов увеличивается. К 2023 г. врачи практически каждого субъекта России обращаются в ГНЦ РФ ФГБУ «НМИЦ эндокринологии» за ТМК по профилю «эндокринология» и «детская эндокринология». Количество консультируемых регионов возросло до 88, включая вновь присоединенные территории (рис. 1).

К 2023 г. к списку регионов, проводящих консультации с применением телемедицинских технологий с Центром, добавились Республика Ингушетия, Луганская Народная Республика, Республика Тыва, Донецкая Народная Республика, Московская область, Запорожская область, Томская область, Херсонская область, город Санкт-Петербург и Республика Алтай.

Количество телемедицинских консультаций «врач-врач»

Ежегодно спрос на ТМК с Центром по профилю «эндокринология» и «детская эндокринология» увеличивается. В 2019 г. число проведенных ТМК между регионами и НМИЦ эндокринологии составило 1166, в 2020 г. — 1837, в 2021 г. — 2923, в 2022 г. — 3824, в 2023 г. — 4725. (рис. 2).

Традиционно превалирует число плановых ТМК по сравнению с неотложными. В 2019 г. количество плановых ТМК составило 1038, в 2020 г. — 1638, в 2021 г. — 2553, в 2022 г. — 3500, в 2023 г. — 4598. В то время как число неотложных консультаций в 2019 г. — 64, в 2020 г. — 55, в 2021 г. — 218, в 2022 г. — 206, в 2023 г. — 127 соответственно.

Регионы-лидеры по частоте обращаемости за ТМК

Анализ данных по обращаемости медицинских сотрудников за ТМК в Центр по профилям «эндокринология» и «детская эндокринология» позволил выделить регионы-лидеры. Начиная с 2020 г. наибольшее количество обращений за ТМК у Ямало-Ненецкого автономного округа (ЯНАО). Ежегодно число ТМК с субъектом растет: в 2019 г. состоялась 41 консультация, в 2020 г. — 103, в 2021 г. — 228, в 2022 г. — 253, в 2023 г. — 321.

В 2019 г. список лидеров по обращаемости состоял из Челябинской области (99 консультаций), Тамбовской области (70 консультаций), Астраханской области (49 консультаций). В 2020 г., помимо ЯНАО (103 консультации), в лидеры вошли Нижегородская (81 консультация) и Челябинская области (72 консультации). В 2021 г. к ЯНАО (228 консультаций) присоединилась Астраханская область (114 консультаций) и Камчатский край (96 консультаций). В 2022 г. — ЯНАО (253 консультации), Тамбовская область (174 консультации) и Астраханская область (159 консультации). В 2023 г. — традиционно — ЯНАО (321 консультация), Астраханская область (183 консультации) и Ханты-Мансийский автономный округ Югра (150 консультаций).

Таким образом, наиболее активно за ТМК в формате «врач-врач» для пациентов взрослого и детского возраста в НМИЦ эндокринологии обращались врачи из Ямало-Ненецкого автономного округа, Тамбовской области и Астраханской области (рис. 3).

Нозологическая структура

Наибольшее количество дистанционных консультаций «врач-врач» пришлось на 2022 и 2023 гг. В связи с этим проведен анализ нозологической структуры обращаемости за медицинской помощью с применением телемедицинских технологий именно за этот период.

В 2022 г. нозологическая структура состояла из 257 диагнозов, в 2023-м — из 208 диагнозов, направленных в рамках ТМК (табл. 1). Среди общего перечня выделены нозологии, по поводу которых консультировали чаще всего по профилям «эндокринология» и «детская эндокринология» в 2022 и 2023 гг. соответственно (рис. 4).

Межинститутские взаимодействия

В рамках работы отдела по вопросам телекоммуникаций Центр активно взаимодействует с другими федеральными центрами. Коморбидные пациенты, наблюдаемые в эндокринологическом учреждении, зачастую требуют привлечения опыта и знаний врачей смежных специальностей. С 2019 года наблюдается устойчивая динамика роста взаимодействия ГНЦ РФ ФГБУ «НМИЦ эндокринологии» с другими федеральными учреждениями (рис. 5).

Госпитализация по результатам ТМК

Один из возможных исходов после проведенной ТМК между Центром и регионом, — госпитализация пациента в федеральное учреждение. Основные цели госпитализации — уточнение диагноза, выявление осложнений, в том числе выполнение диагностических тестов, проведение оперативного лечения при необходимости. С каждым годом наблюдается тенденция к увеличению данного показателя. Так, в 2023 г. было госпитализировано 1052 пациента в НМИЦ эндокринологии по результатам ТМК (рис. 6).

В 2023 г. отмечена высокая активность взаимодействия новых территорий (ДНР, ЛНР, Херсонская и Запорожская области) с ГНЦ РФ ФГБУ «НМИЦ эндокринологии». В рамках дистанционного взаимодействия специалисты из вышеперечисленных субъектов направили 95 запросов на ТМК, по результатам которых 21 пациент был госпитализирован в Центр, что составляет 20% от общего количества.

**Figure:**
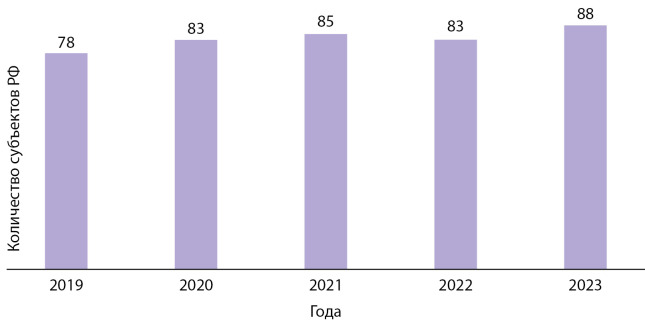
Рисунок 1. Сравнительная динамика обращаемости регионов за ТМК.

**Figure:**
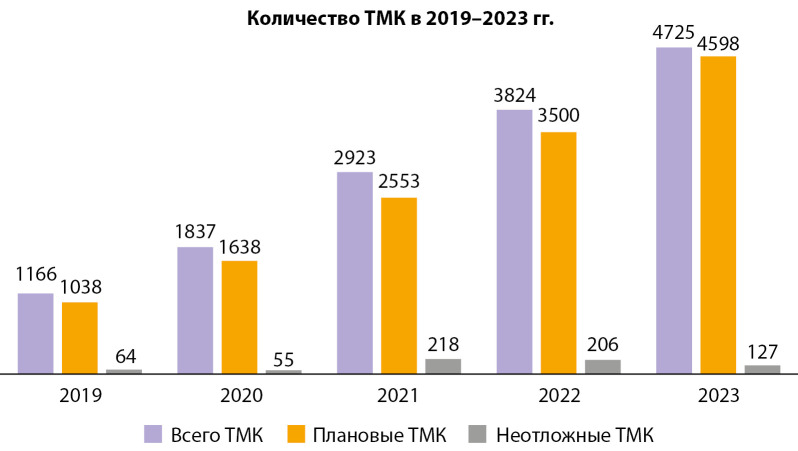
Рисунок 2. Распределение проведенных ТМК между регионами и Центром за 2019–2023 гг.

**Figure:**
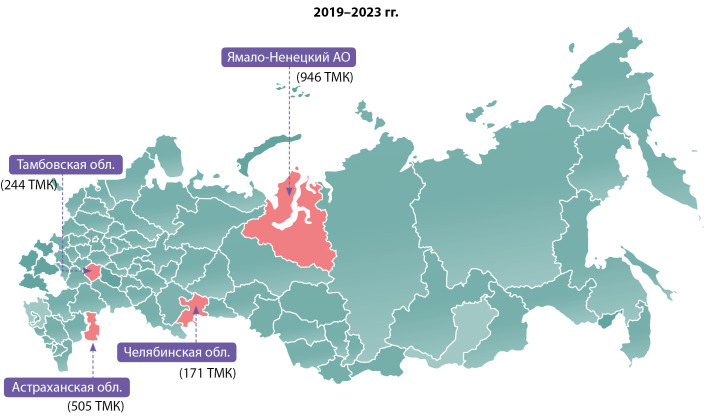
Рисунок 3. Распределение регионов-лидеров по количеству запросов на ТМК по профилям «эндокринология» и «детская эндокринология» с количественным указанием проведенных с ними ТМК за 2019–2023 гг.

**Figure:**
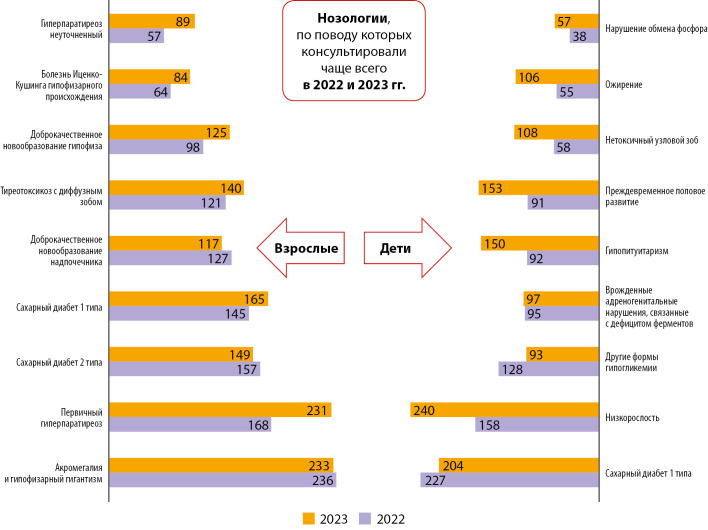
Рисунок 4. Нозологии, по поводу которых консультировали чаще всего взрослых и детей в 2022 и 2023 гг.

**Figure:**
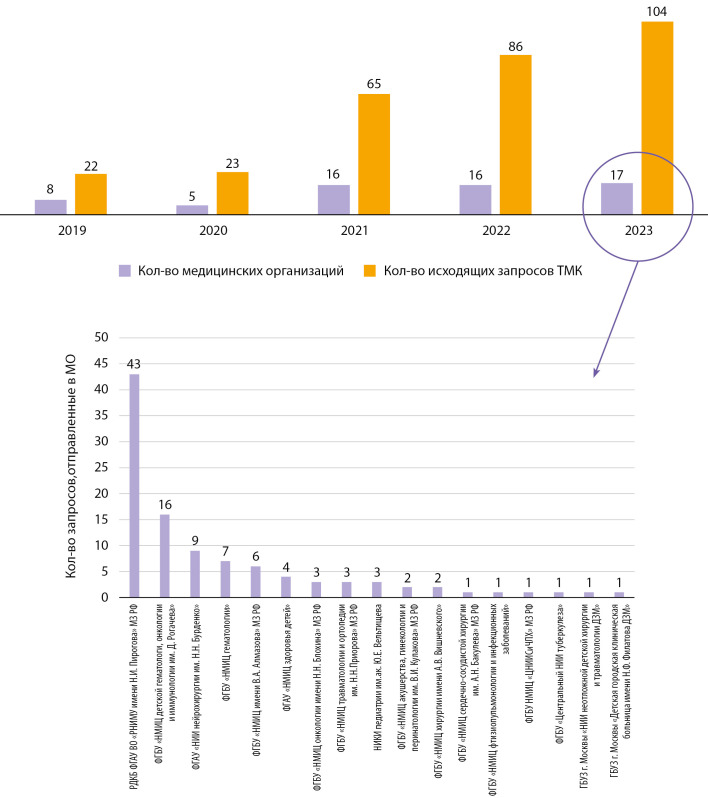
Рисунок 5. Динамика взаимодействия ГНЦ РФ ФГБУ «НМИЦ эндокринологии» с другими федеральными центрами, а также показатели «исходящих запросов», отправленные Центром через систему ФТМС в 2019–2023 гг.

**Figure:**
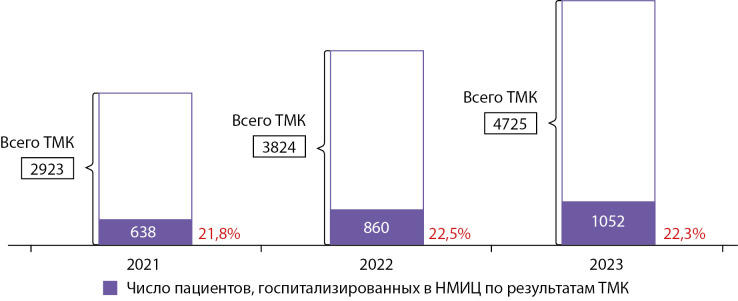
Рисунок 6. Динамика госпитализированных пациентов по результатам ТМК 2021–2023.

**Table:** Таблица 1. Перечень всех нозологий, по которым были направлены запросы на ТМК «врач-врач» в 2022 и 2023 гг. Диагнозы указаны в соответствии с кодами МКБ-10.

№	Направительные диагнозы для ТМК «врач-врач» (взрослые и дети)	Кол-во за 2022 г.	Кол-во за 2023 г.
1	E10-E14: Сахарный диабет	622	671
2	E21: Гиперпаратиреоз	287	406
3	E22.0: Акромегалия и гипофизарный гигантизм	238	237
4	D35.0: Новообразования надпочечника	218	139
5	E05: Тиреотоксикоз	215	252
6	E16: Гипогликемии	209	209
7	E34.3: Низкорослость	182	281
8	E23.0: Гипопитуитаризм	174	232
9	E04: Нетоксический зоб	160	215
10	E25: Адреногенитальные расстройства	158	160
11	E24: Синдром Иценко-Кушинга	139	155
12	E22: Гиперфункция гипофиза	121	156
13	E66-68: Ожирение и другие виды избыточности питания	116	196
14	E83: Нарушение минерального обмена	108	128
15	D35.2: Доброкачественное новообразование гипофиза	106	132
16	E30: Нарушение полового созревания	102	153
17	Q: Врожденные аномалии [пороки развития], деформации и хромосомные нарушения	82	94
18	E89: Эндокринные и метаболические нарушения, возникшие после медицинских процедур	79	106
19	E27: Другие нарушения надпочечников	77	205
20	C73-C75: Злокачественное новообразование щитовидной железы и других эндокринных желез	67	63
21	E31: Полигландулярная дисфункция	56	58
22	E26: Гиперальдостеронизм	48	53
23	O: Состояния, связанные с беременностью	44	1
24	R,T,Z,A,B,G,F, J: Иное	36	51
25	E20: Гипопаратиреоз	32	33
26	E29: Дисфункция яичек	30	32
27	E06: Тиреоидит	29	31
28	I: Болезни системы кровообращения	26	40
29	M80-85: Нарушения плотности и структуры кости	26	43
36	E70-88: Нарушение обмена веществ	23	29
30	E28: Дисфункция яичников	21	28
31	E23.2: Несахарный диабет	19	32
32	E03: Другие формы гипотиреоза	14	42
33	C00-97: Другие злокачественные образования	15	3
34	E34: Другие эндокринные нарушения	15	24
35	N: Болезни мочеполовой системы	13	17
36	D: Другие новообразования	12	20
37	E50-64: Другие виды недостаточности питания	13	12
38	E07: Другие болезни щитовидной железы	6	7
39	P: Состояния, возникающие в перинатальном периоде	6	7
40	E43-46: Белково-энергетическая недостаточность	5	22
41	D34: Доброкачественное новообразование щитовидной железы	4	11
42	L: Болезни кожи	3	3
43	E01: Болезни щитовидной железы, связанные с йодной недостаточностью, и сходные состояния	1	2
44	M86-90: Другие остеопатии	-	5
45	E32.8: Другие болезни вилочковой железы	-	2
46	D35.1: Доброкачественное новообразование паращитовидной [околощитовидной] железы	-	1

## Обсуждение

## Репрезентативность выборок

Анализ телемедицинских консультаций в формате «врач-врач» проводился только в разрезе работы ГНЦ РФ ФГБУ «НМИЦ эндокринологии», что ограничивает репрезентативность полученной выборки и демонстрирует опыт оказания медицинской помощи с применением телемедицинских технологий на примере одного федерального учреждения.

## Сопоставление с другими публикациями

Полученные данные демонстрируют эффективность телемедицинской помощи в настоящее время. Дистанционное взаимодействие позволяет получать консультации и рекомендации узкопрофильных специалистов высокого уровня, что обеспечивает оптимизацию лечебного процесса, улучшая качество оказываемой помощи.

Анализ территориальной структуры обращаемости за телемедицинскими консультациями в рамках «врач-врач» за 2019–2023 г. указывает на ежегодное расширение охваченных ТМК границ. В 2023 г. врач практически каждого уголка России получил помощь в диагностике и лечении пациентов по профилям «эндокринология» и «детская эндокринология».

Причина увеличения числа консультируемых регионов — в растущей осведомленности медицинских сотрудников о возможностях ТМК с федеральными учреждениями. Немалый вклад вносят выездные мероприятия ГНЦ ФГБУ «НМИЦ эндокринологии» в субъекты Российской Федерации и выполняемая врачами Центра просветительская работа в рамках выезда.

Техническая составляющая в виде обеспечения медицинских сотрудников субъектов РФ доступа к интернету на рабочем месте также играет важную роль в формировании запроса на ТМК. Повышается и информированность пациентов, проходивших лечение в НМИЦ эндокринологии. Выписные эпикризы содержат исчерпывающие сведения для лечащего врача в регионе о возможности дальнейшего наблюдения в Центре с помощью ТМК.

Регион-лидер по частоте обращаемости за ТМК — Ямало-Ненецкий автономный округ — характеризуется высокой территориальной разрозненностью. Большие расстояния между лечебно-профилактическими учреждениями ставят пациентов в затруднительное положение при необходимости обследования и лечения. Вклад в рост числа запросов из региона вносит и финансирование ТМК за счет ФОМС.

Одним из важных вопросов является анализ нозологической структуры обращаемости за телемедицинской помощью по направлению «врач-врач». За 2022–2023 гг. наиболее популярными направительными нозологиями являлись «сахарный диабет» (СД) и «гиперпаратиреоз» (табл. 1).

При детальной оценке в разрезе возрастной структуры у взрослых наибольшее количество ТМК было проведено по поводу акромегалии и гипофизарного гигантизма, а также первичного гиперпаратиреоза. Высокая обращаемость по данным нозологиям обусловлена несколько аспектами.

Акромегалия является тяжелым нейроэндокринным заболеванием, для лечения которого необходима мультидисциплинарная команда с возможностью проведения хирургического лечения. Во многих регионах отсутствуют отделения эндокринной хирургии с возможностью выполнения нейрохирургических вмешательств. Ввиду невысокой распространенности заболевания отсутствует должный опыт проведения транссфеноидальных аденомэктомий, назначения эндокринологами медикаментозного лечения и должного динамического наблюдения.

В отношении патологии минерального обмена популярность проведения ТМК по поводу первичного гиперпаратиреоза связана как с достаточной распространенностью (нозология занимает 3-е место по распространенности эндокринологических патологий после сахарного диабета и патологии щитовидной железы) [5–6], так и со сложностями установки точного диагноза и определения тактики ведения. Требуемые знания и клинический опыт введения пациентов с первичным гиперпаратиреозом (диагностические пробы с целью дифференциальной диагностики форм гиперпаратиреоза, возможности проведения различной топической диагностики образований околощитовидных желез, выполнение хирургического лечения) ограничивают ведение таких пациентов в ряде регионов, вследствие чего врачи обращаются за экспертным мнением в ГНЦ РФ ФГБУ «НМИЦ эндокринологии».

В части детской эндокринологии популярными направительными диагнозами в рамках ТМК являлись сахарный диабет 1 типа (СД1) и низкорослость. Превалирующее число дистанционных консультаций по СД было связано с решением вопроса о госпитализации в федеральный центр в случаях, требующих уточнения диагноза, проведения молекулярно-генетического исследования, установки или замены помповой инсулинотерапии, средств непрерывного мониторирования глюкозы, а также прохождения структурированного обучения для детей и их законных представителей.

Задержка роста является частой причиной обращения к детскому эндокринологу. Поиск этиологического фактора, проведение дифференциальной диагностики, инициация медикаментозной терапии гормоном роста — требуют экспертной оценки и консультации специалистов ГНЦ РФ ФГБУ «НМИЦ эндокринологии».

Также вызывает интерес анализ не только «входящих» ТМК консультаций, поступающих в Центр, но и оформление «исходящих запросов» Центром в другие медицинские организации (МО) 3 уровня. ГНЦ РФ ФГБУ «НМИЦ эндокринологии» активно сотрудничает с другими МО. Рисунок 8 отражает увеличивающуюся динамику как по количеству взаимодействующих МО, так и по общему числу «исходящих запросов» за последние 5 лет. Наиболее активный контакт отмечен с Российской детской клинической больницей (филиала ФГАОУ ВО РНИМУ им. Н.И. Пирогова Минздрава России). Данная корреляция обусловлена, вероятнее всего, с тяжестью детей, поступающих в Институт детской эндокринологи ГНЦ РФ ФГБУ «НМИЦ эндокринологии» и требующих консультаций междисциплинарной команды педиатров и специалистов детского профиля.

Госпитализация в стационар является одним из результатов проведения ТМК. С 2021–2023 гг. около 20% пациентов были госпитализированы в ГНЦ РФ ФГБУ «НМИЦ эндокринологии» после проведенных дистанционных консультаций (рис. 3). Стоит отметить, что данный процент сохранялся и по ТМК, полученным с новых территорий (ДНР, ЛНР, Херсонская и Запорожская области).

## Клиническая значимость результатов

Согласно нашим данным, дистанционные консультации в формате «врач-врач» являются удобным и эффективным способом оказания медицинской помощи с применением телемедицинских технологий.

## Ограничения исследования

Исследование проводилось впервые и имеет ряд ограничений. В первую очередь, оценка дистанционных консультаций проводилась только по профилям «эндокринология» и детская эндокринология». Анализ оказания медицинской помощи с применением телемедицинских технологий проводили только на базе ГНЦ РФ ФГБУ «НМИЦ эндокринологии», что ограничивает репрезентативность выборки.

## Направления дальнейших исследований

Консультирование с применением телемедицинских технологий является удобным и перспективным способом оказания медицинской помощи, а также полезным инструментом взаимодействия врачебного сообщества. Необходимо проведение многоцентровых исследований с целью оценки эффективности ТМК по разным направлениям, рассмотрение вопроса выполнения ТМК в субъектах РФ в рамках ОМС.

## Заключение

Усовершенствование дистанционных технологий и полученный опыт организации медицинской помощи во время пандемии COVID-19 способствовали развитию телемедицинской помощи в РФ.

Телекоммуникационное взаимодействие позволяет врачам из различных медицинских организаций консультироваться по большому количеству вопросов, возникающих при ведении пациентов с эндокринопатиями, охватывая широкий спектр различных нозологий и патологий. Чаще всего при формировании телемедицинского запроса врачи обращаются с целью уточнения диагноза, получения рекомендаций по проведению дополнительных обследований, в том числе решения вопроса о выполнении генетического исследования, маршрутизации пациента в федеральный центр, а также динамического наблюдения пациентов, ранее проходивших лечение в ГНЦ РФ ФГБУ «НМИЦ эндокринологии».

Телемедицина также выступает в роли инструмента оценки уровня медицинской помощи в различных субъектах РФ, в том числе демонстрируя вовлеченность врачей в данный вид взаимодействия. Анализ качества и содержания телемедицинских запросов, поступающих из субъектов РФ, помогает сделать вывод об уровне медицинской помощи в данном субъекте РФ.

Таким образом, немаловажно популяризировать телемедицинское направление, активно внедрять в практическое здравоохранение и расширять функционал оказания дистанционной медицинской помощи.

## Дополнительная информация

Источники финансирования. Работа выполнена по инициативе авторов без привлечения финансирования.

Конфликт интересов. Авторы декларируют отсутствие явных и потенциальных конфликтов интересов, связанных с содержанием настоящей статьи.

Участие авторов. Назарова А.С. — концепция и дизайн исследования, получение, анализ данных, интерпретация результатов, написание рукописи; Приказчикова С.С. — концепция и дизайн исследования, написание статьи; Калашников В.Ю., Мельниченко Г.А., Мокрышева Н.Г. — концепция и дизайн исследования, внесение в рукопись существенных правок с целью повышения научной ценности статьи. Все авторы одобрили финальную версию статьи перед публикацией, выразили согласие нести ответственность за все аспекты работы, подразумевающую надлежащее изучение и решение вопросов, связанных с точностью или добросовестностью любой части работы.
